# Crosstalk between RON and androgen receptor signaling in the development of castration resistant prostate cancer

**DOI:** 10.18632/oncotarget.7287

**Published:** 2016-02-09

**Authors:** Izhar Batth, Huiyoung Yun, Suleman Hussain, Peng Meng, Powel Osumulski, Tim Hui-Ming Huang, Roble Bedolla, Amanda Profit, Robert Reddick, Addanki Kumar

**Affiliations:** ^1^ Department of Urology, San Antonio, TX, USA; ^2^ Department of Pharmacology, San Antonio, TX, USA; ^3^ Department of Molecular Medicine, San Antonio, TX, USA; ^4^ Department of Pathology, San Antonio, TX, USA; ^5^ Department of Cancer Therapy and Research Center, San Antonio, TX, USA; ^6^ The University of Texas Health Science Center at San Antonio and South Texas Veterans Health Care System, San Antonio, TX, USA; ^7^ Current address: Life Sciences Division, Lawrence Berkley National Laboratory, Berkley, CA, USA; ^8^ Current address: Department of Pediatrics, The University of Texas MD Anderson Cancer Center, Houston, TX, USA

**Keywords:** castrate resistant prostate cancer, apoptosis, FLIP, RON, MST1R

## Abstract

Castrate-resistant prostate cancer (CRPC) is the fatal form of prostate cancer. Although reactivation of androgen receptor (AR) occurs following androgen deprivation, the precise mechanism involved is unclear. Here we show that the receptor tyrosine kinase, RON alters mechanical properties of cells to influence epithelial to mesenchymal transition and functions as a transcription factor to differentially regulate AR signaling. RON inhibits AR activation and subset of AR-regulated transcripts in androgen responsive LNCaP cells. However in C4-2B, a castrate-resistant sub-line of LNCaP and AR-negative androgen independent DU145 cells, RON activates subset of AR-regulated transcripts. Expression of AR in PC-3 cells leads to activation of RON under androgen deprivation but not under androgen proficient conditions implicating a role for RON in androgen independence. Consistently, RON expression is significantly elevated in castrate resistant prostate tumors. Taken together our results suggest that RON activation could aid in promoting androgen independence and that inhibition of RON in combination with AR antagonist(s) merits serious consideration as a therapeutic option during hormone deprivation therapy.

## INTRODUCTION

In men, prostate cancer (PCA) is the most common cancer and the second leading cause of cancer deaths in the United States [1]. Development of PCA progresses from androgen-dependent to androgen-independent and hormone-refractory metastatic state. Growth and development of prostate depends on androgens, therefore androgen deprivation therapy (ADT) such as androgen antagonists and androgen synthesis inhibitors are used as therapeutic strategies [2]. Although ADT is initially effective; PCA inevitably re-emerges as aggressive metastatic castrate-resistant prostate cancer (CRPC) [3]. Reactivation of androgen receptor (AR) signaling axis through modulation of AR co-activator/co-repressors, non-androgenic hormones or receptor tyrosine kinases (RTKs) are likely reasons for progression to CRPC [4, 5]. However, to the best of our knowledge there is no effective curative option for metastatic CRPC [6]. Therefore, it is paramount that the transformation of this disease from a localized to metastatic castrate-resistant state be further scrutinized for possible therapeutic opportunities.

The recepteur d'origine nantais (RON), also known as macrophage stimulating-1 receptor (MST1R), is an RTK which shares structural similarities with c-Met [7]. Upon activation by its ligand macrophage stimulating protein (MSP), RON undergoes either homo or heterodimerization with other RTKs including MET, EGFR, PDGFR, and IGF1R to exert its biological activity [8–14]. Aberrant expression of RON either as a result of its overexpression or constitutive activation has been reported in many tumor types including pancreas, liver, breast, colon, ovarian, prostate, and bladder [9, 15–25]. Further, RON overexpression is associated with tumor metastasis and shorter patient survival suggesting a role in promoting tumor progression [15]. Nevertheless, compared to other tumor types, role of RON in prostate carcinogenesis is not well studied. Therefore, understanding how RON contributes to prostate cancer progression may guide therapeutic strategies to inhibit progression to metastatic CRPC.

Here we show that RON levels and expression are significantly elevated in (i) high-grade and metastatic castrate resistant human prostate tumors and (ii) advanced prostate cancer cells. Overexpression and knockdown studies demonstrate that RON promotes epithelial mesenchymal transition (EMT) and decreases cell adhesion and increasing elasticity. More importantly, ectopic expression of RON has contrasting effects on AR signaling in AR-positive and AR negative cells. Further, RON can function as a transcription factor to regulate c-FLIP possibly in a context or cell-dependent manner. Remarkably, RON is activated as an alternate by-pass signaling mechanism to compensate for loss of AR under androgen deprived conditions. These observations lend credence to our hypothesis that activation of RON under androgen-deprived conditions activates AR pathway leading to castrate-resistance.

## RESULTS

### Elevated levels and expression of RON in prostate tumors

**Figure 1:**
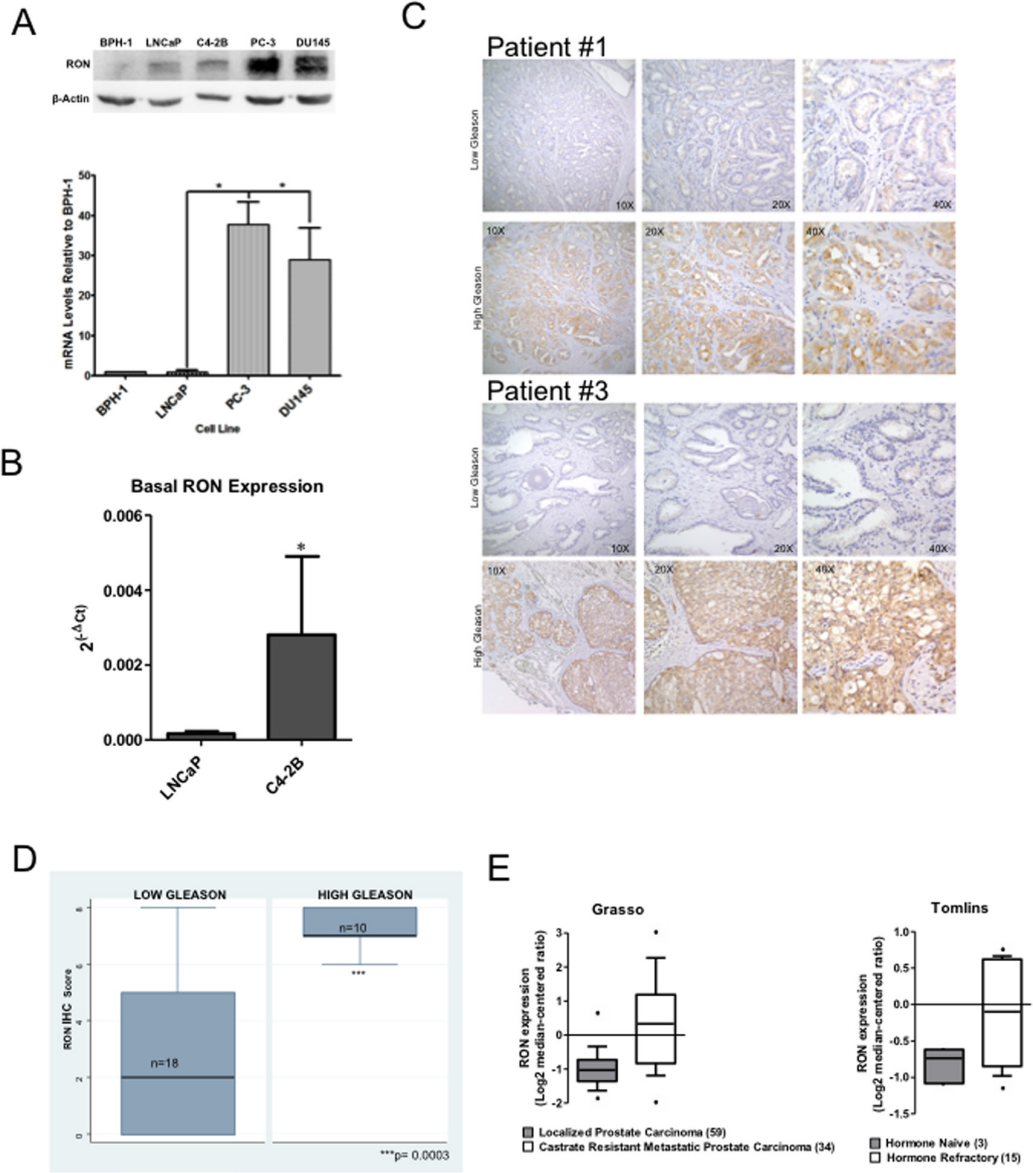
Elevated levels of RON in human prostate cancer cells and tumors Whole cell extracts and total RNA prepared from non-tumorigenic BPH-1, androgen responsive LNCaP, C4-2B, a castrate-resistant sub line of LNCaP, androgen independent PC-3 and DU145 cells was used in immunoblot analysis and real-time PCR using RON-specific primers. **A.** A representative immunoblot gel (top panel) and RON expression changes (average+sd) normalized to endogenous β-actin relative to BPH-1 cells (bottom panel) from three independent experiments (n=2 for western blot panel and n=4 for mRNA expression each with three technical replicates) is shown. **B.** RON expression changes (average+sd) normalized to endogenous β-actin in androgen-sensitive LNCaP (n=9 biological replicates each with 3 technical replicates) and castrate-resistant C4-2B (n=6 biological replicates each with 3 technical replicates) cells. Statistical significance was determined using two-sided t-test and with no adjustment for multiple comparisons. **C.** Tissue microarray containing low (Gleason score ≤ 6; n=18 biological replicates) and high Gleason grade (Gleason score ≥ 7; n=10 biological replicates) human prostate tumor specimens were stained using RON polyclonal antibody at a dilution of 1:50. A representative immunohistochemical staining of RON expression of low and high Gleason from two different patients (#1 and 2) is shown. **D.** TMAs were scored semi-quantitatively as described by us previously based on the proportion (percent) and intensity (negative, 1+ for low, 2+ for medium and 3+ for high). Final staining score was obtained as product of proportion and intensity of staining. Box plot showing differential expression of RON in low and high Gleason tumors. Wilcoxon (Mann-Whitney U) test was performed to determine if the mean ranks of RON total scores differed among tissues grouped by low Gleason of 4 or 6 (n=18) vs. High Gleason of 7 to 10 (n=10). The groups were also compared with a T test allowing for unequal variances with a Welch approximation with similar results but the non-parametrical test was considered the best fit for the data (STATA 9.2). **E.** Expression changes of RON in localized and castrate resistant metastatic prostate tumors. Data extracted from Oncomine.

We examined the levels and expression of RON using immortalized human prostate epithelial cell line (BPH-1), androgen-responsive LNCaP, and androgen independent PC-3 and DU145. RON levels and expression were significantly elevated in PC-3 and DU145 compared to LNCaP cells (Figure 1A). In addition castrate-resistant C4-2B cells exhibited significantly elevated RON expression relative to its parental LNCaP cells (Figure 1B). Although RON expression is elevated in other tumor cell lines such as melanoma and bladder compared to their non-tumorigenic counter parts, the expression is much higher in prostate cancer cells (Supplementary Figure 1). We used immunohistochemistry to analyze the expression of RON as a function of the grade of human prostate tumors. We found significantly (p=0.0003) higher staining of RON in high Gleason grade (7 to 10) compared to low Gleason grade (4 or 6) tumors (Figure 1C and 1D). Additionally, analysis of Oncomine data showed RON expression was significantly elevated in metastatic castrate resistant human prostate tumors (Figure 1E). Taken together, these observations suggest that RON levels and expression are significantly elevated in advanced prostate cancer cells, high-grade and castrate resistant human prostate tumors.

### RON modulates mechanical properties of prostate cancer cells

**Figure 2:**
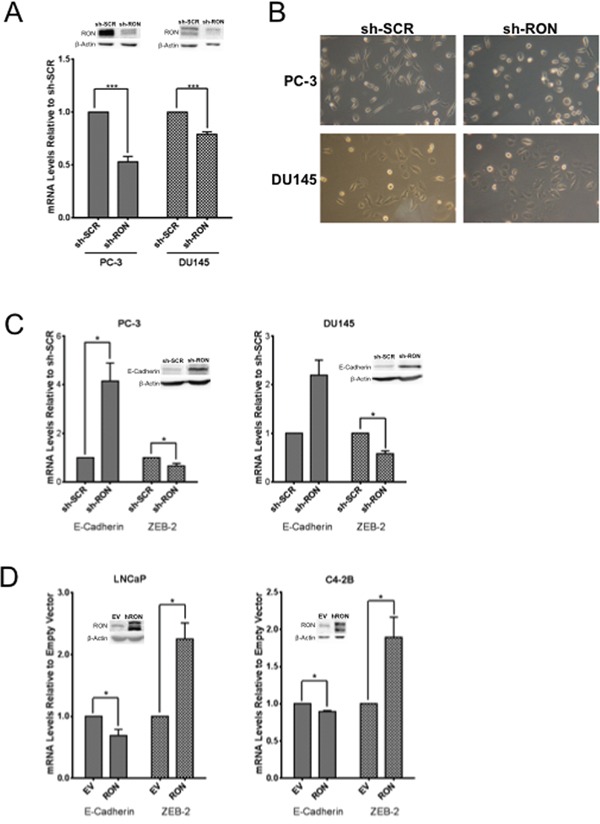
RON contributes to epithelial mesenchymal transition Whole cell extracts and total RNA was extracted from logarithmically growing PC-3 (n= 4 biological replicates) or DU145 (n= 3 biological replicates) cells stably silenced with RON-specific shRNA or scrambled shRNA and used in **A.** validation of knockdown, **B.** morphological alterations, **C.** alterations in epithelial (E-cadherin) and mesenchymal (ZEB-2) markers (n=2 biological replicates each with triplicate replicates). **D.** 48 h after transfection, whole cell extracts and total RNA was prepared from androgen responsive LNCaP (n= 4 biological replicates with triplicate technical replicates) and C4-2B (n= 3 biological replicates with triplicate technical replicates), a castrate-resistant sub line of LNCaP cells transiently transfected with RON cDNA was used in analyzing expression of epithelial (E-cadherin) and mesenchymal (ZEB-2) markers. Data presented is an average+sd of three independent experiments. Statistical significance of the data was determined using students t-test and p<0.05 was considered significant. EV = Cells transfected with empty vector and RON, transfected with RON cDNA expression plasmid.

**Figure 3:**
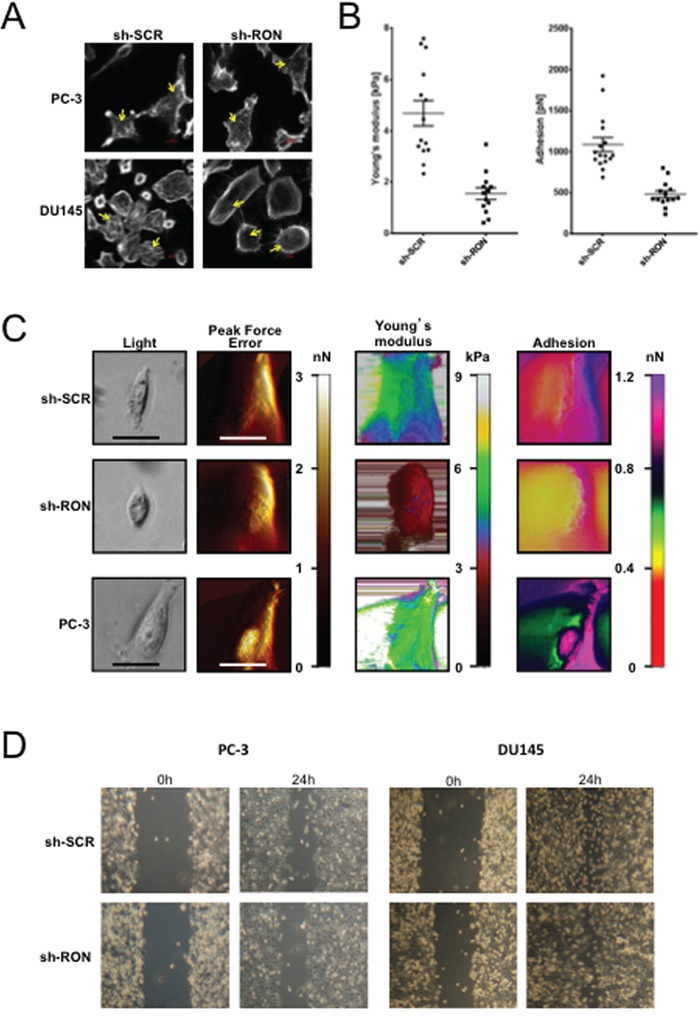
RON regulates mechanical properties of cells **A.** Logarithmically growing stable RON-KD or non-targeted PC-3 (n= 3 biological replicates) or DU145 (n= 3 biological replicates) cells were stained for F-actin using Rhodamine-phalloidin. Images were captured using a Sweptfield confocal system equipped with a Nikon Ti microscope at 60X magnification. An arrow indicates differences in F-actin organization. A representative image from three independent experiments is shown. **B.** Elasticity expressed, as the Young's modulus in kPa and adhesion expressed in Newtons was determined for at least 40 RON-KD PC-3 cells or non-targeted controls using atomic force microscopy. Data was normally distributed. Means between two groups were compared using unpaired t-test (Welch correction) and outliers detected with the ROUT or Grubbs methods (Graph Pad Prism and OriginLab Pro 9.1), p<0.05 was considered statistically significant. **C.** Representative images obtained with the Peak Force QNM AFM showing distinct nanomechanical properties (light microscopy image, peak force error (edge detection and fine topographical details)), cell elasticity (Young's modulus, kPa) and cell adhesion (nN) of stably silenced RON (n= 14 biological replicates) or non-targeted PC-3 (n= 16 biological replicates) or wild type PC-3 (n= 9 biological replicates) cells. Peak Force Error images were used to determine location of the cell boundary collected in elasticity and adhesion channels. All the images (except light microscopy) are false colored. The Peak Force Error scale shows smaller to taller objects progressing from black to white color. The Young's modulus (elasticity) scale shows softer objects as black and brown (lower modulus) and more rigid as green and yellow (higher modulus). The adhesion scale shows less adhesive objects as yellow and green (less force needed to separate an AFM tip from a cell) and stickier objects as dark blue and pink (more force needed). The black and white scale bars represent 40 and 20 μm, respectively. **D.** Photographs of gap closure following wound scratch of monolayer cells monitored every 6h. The experiment was repeated six times for PC-3 (n=6) and thrice for DU145 (n= 3) and a representative phase contrast image from an inverted Zeiss Primo Vert light microscope is shown.

To examine the functional significance of RON in prostate cancer we stably knocked down (KD) RON in PC-3 and DU145 cells. These cells showed consistent decrease in mRNA and protein levels compared to non-targeted control cells (Figure 2A). Microscopy observations revealed PC-3-RON-KD cells were flat with epithelial like morphology, while non-targeted cells expressing RON retained original spindle shaped mesenchymal appearance (Figure 2B and Supplementary Figure 2 for higher magnification image). Based on these findings we examined alterations in the expression and levels of EMT related genes. We found that RON-KD led to significant increase in E-cadherin and decrease in ZEB-2 expression in both PC-3 and DU145 cells (Figure 2C). Additionally, RON overexpression in LNCaP and its castrate-resistant sub line C4-2B cells led to robust increase in ZEB-2 expression and a marginal but significant decrease in E-cadherin (Figure 2D). EMT changes are associated with actin cytoskeleton reorganization, and tumor cells are at an advantage to migrate and adhere because of their enhanced elasticity (smooth surface) compared to non-tumorigenic cells [26, 27]. To investigate the functional significance of the observed RON-induced changes on EMT we examined cytoskeletal reorganization using phalloidin staining. We found that RON-KD cells had disorganized actin stress fibers indicated by lack of spindle fibers and reduced intensity of phalloidin staining; while such changes were not evident in non-targeted control cells (Figure 3A). Next we used atomic force microscopy (AFM) to determine changes of mechanical properties typical of EMT such as enhanced cell elasticity and decreased adhesive capacity [28]. Elasticity measures the capacity of cells to reversibly resist deformation. It is expressed in units of pressure (Pascals, Pa) as the Young's modulus. More elastic and softer cells have lower Young's moduli. Elasticity is a complex result of interplay of cytoskeleton properties, membrane fluidity, cytoplasm density and distribution of organelles. On the other hand, cell adhesion quantifies the capability of a cell to adhere to another object. Adhesion is expressed in units of force as Newtons (N) and its higher values correspond to more adherent cells. Adhesion is a product of chemical properties of a cell membrane including lipid rafts and type and distribution of membrane proteins. AFM results show that non-targeted PC-3 cells exhibited a mean elasticity of 4.68 kPa and mean adhesion of 1089 pN (Figure 3B). In contrast, RON-KD PC-3 cells were about 3 times less rigid and about 2 times less adhesive. Therefore, we conclude that the RON-KD cells showed changes in the mechanical phenotype consistent with EMT [29–31]. Light microscopy and AFM images comparing morphological and mechanical properties of representative cells are shown in Figure 3C. Finally we used wound closure assay as a measure of migration. Interestingly, RON depletion had no significant effect on *in vitro* migratory ability of PC-3 or DU145 cells (Figure 3D). These data suggest that RON is elevated in androgen-independent prostate cancer cells and could contribute to cytoskeletal and mechanical properties of cells associated with EMT. In addition, RON *per se* may not be involved in prostate cancer cell migration as opposed to other types of tumor cells. Studies to determine whether RON targets these EMT markers directly or indirectly are in progress in our laboratory.

### RON has differential effects on AR and its target genes in androgen responsive and castrate-resistant cells

**Figure 4:**
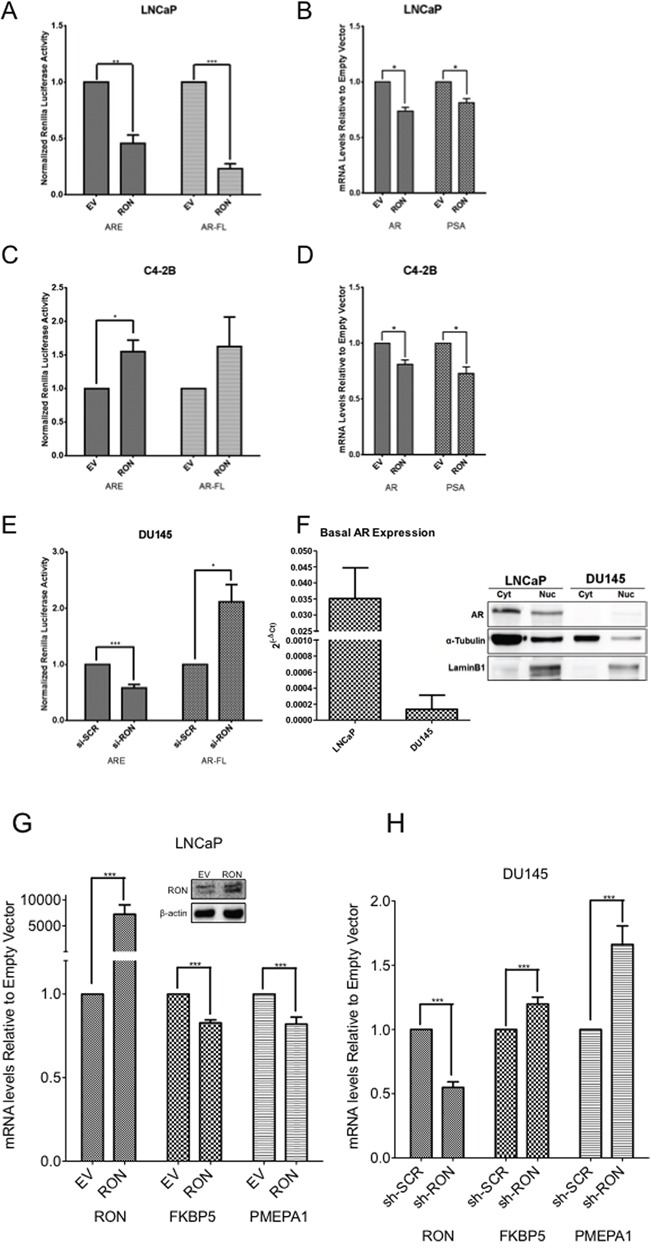
RON suppresses native AR but activates ARE **A-F.** Reporter plasmids including pGL3-ARE containing three copies of PSA AREs and AR-reporter (1.7kb) construct containing the firefly luciferase gene were co-transfected with empty vector pcDNA 3.1 (EV) or RON expression plasmid (RON) into androgen responsive LNCaP (A:ARE n= 3 and AR n=6 biological replicates with triplicate technical replicates) and (B:AR n= 3, PSA n= 4 biological replicates with triplicate technical replicates) and castrate-resistant LNCaP sub line C4-2B cells (C:ARE n= 3 and AR n= 2 biological replicates with triplicate technical replicates) and (D:AR and PSA n= 4 biological replicates with triplicate technical replicates) together with Renilla luciferase. 24h after transfection, luciferase activity in cell lysates was measured (A and C). Normalized luciferase/renilla activity was calculated with respect to EV. Data shown are average+sd of three independent experiments conducted in triplicate. Total RNA was prepared from androgen responsive LNCaP and castrate-resistant LNCaP sub line C4-2B cells transiently transfected with empty vector pcDNA 3.1 (EV) or RON expression plasmid (RON) was used for measuring changes in expression of AR and PSA (B and D). E. pGL3-ARE containing three copies of PSA AREs and full-length 1.7kb AR constructs containing the firefly luciferase were co-transfected with scrambled siRNA or RON siRNA along with renilla luciferase in androgen independent DU145 cells (ARE and AR n= 3 biological replicates with triplicate technical replicates). 48h post-transfection, luciferase activity was measured. Normalized luciferase/renilla activity was calculated with respect to scrambled siRNA. The data shown are average + sd of three independent experiments conducted in triplicate. F. Total RNA, nuclear and cytoplasmic extracts prepared from logarithmically growing LNCaP (n=2 biological replicates each with triplicate technical replicates) and DU145 (n=3 biological replicates each with triplicate technical replicates) cells was used to measure endogenous expression and levels of AR. Statistical significance was determined using two-sided t-test with no adjustment for multiple comparisons. A total of two biological replicates were used for LNCaP and DU145 western blot panel. **G.** Total RNA prepared from LNCaP (n=3 biological replicates each with triplicate replicates) cells transiently transfected with RON cDNA (48h after transfection) was used to analyze endogenous expression of indicated AR-regulated genes. Graphs illustrate relative mRNA quantification relative to empty expression vector as described in the materials and methods section. Data presented is an average+sd of three independent experiments. Statistical significance of the data was determined using students t-test and p<0.05 was considered significant. EV = Cells transfected with empty vector and RON-transfected with RON cDNA expression plasmid. Inset shows overexpression of RON. **H.** Total RNA was extracted from logarithmically growing sh-RON or scrambled DU145 (n=4 biological replicates each with three technical replicates) cells used to analyze endogenous expression of indicated AR-regulated genes. Graphs illustrate relative mRNA quantification relative to non-targeted control as described in the materials and methods section. Significance of the data was determined using students t-test and p<0.05 was considered significant (* = p ≤ 0.05; ** = p ≤ 0.01; *** = p ≤ 0.001).

Androgen-induced EMT changes and cytoskeletal reorganization are reported to be involved in the metastatic behavior of androgen independent prostate cancer cells [32, 33]. To evaluate whether RON mimics androgen-induced changes, we examined the effect of ectopic expression and silencing of RON on AR pathway activation by measuring AR promoter activity and endogenous expression of AR-regulated genes. Our data show that ectopic expression of RON is associated with decreased (i) androgen response element (ARE)-reporter activity (containing three ARE binding sites), and (ii) AR promoter activity in LNCaP cells (Figure 4A). The observed decreased AR promoter activity correlates with decreased mRNA expression of AR and its target gene PSA (Figure 4B). Under similar experimental conditions, we observed increased activity of ARE-reporter in castrate-resistant C4-2B cells (Figure 4C). RON overexpression also reduced AR and PSA expression in C4-2B cells (Figure 4D). These results suggest that transient ectopic expression of RON decreases AR mRNA levels and its established target PSA in AR expressing androgen responsive and castrate-resistant cells. On the other hand, silencing RON in androgen-independent AR-negative DU145 cells resulted in restoration of AR transcriptional activation as assessed by native AR promoter activity (Figure 4E). Surprisingly we detected basal expression of AR mRNA by q-PCR but not protein (Figure 4F). Furthermore, transient overexpression of RON resulted in consistent decrease in mRNA expression of additional AR activated genes including FKBP5 and PMEPA1 in LNCaP cells (Figure 4G). We also observed increased expression of AR-activated genes including PMEPA1 and FKBP5 in DU145 but not in PC-3 cells stably silenced for RON (Figure 4H and data not shown). Consistent with published reports we did not detect expression of AR, PSA or TMPRSS2 in these cells (data not shown). Based on these observations, we speculate that RON may activate a subset of AR target genes in an AR-independent manner in castrate-resistant cells (C4-2B and DU145) (Figure 4C–4E). Our unpublished results also suggest that RON could influence AR and its target gene expression based on the levels of expression. Therefore, we do not rule out the possibility that RON can have differential effects on AR and its target genes in a RON level-dependent manner.

**Figure 5:**
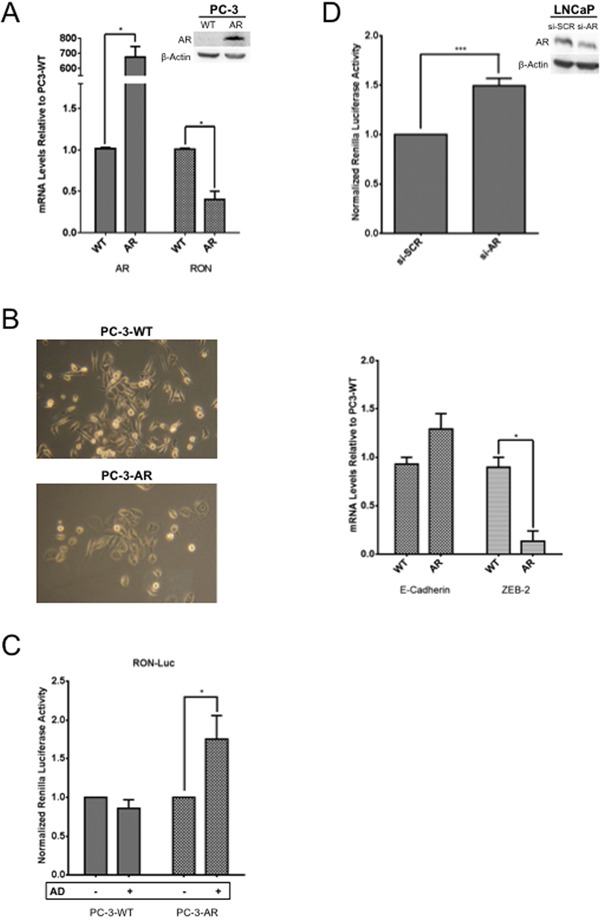
Androgen deprivation, a molecular switch for AR to activate RON **A.** Total RNA, and whole cell extracts prepared from logarithmically growing wild type (PC-3) and stably expressing AR (PC-3AR) cells (n=2 and 3 biological replicates each with triplicate technical replicates for RNA and protein respectively) was used to measure expression and levels of AR and RON. Expression changes (average + sd) in PC-3 AR cells relative to wild type PC-3 cells are shown. **B.** Phase contrast images depicting morphological differences between PC-3AR and wild type PC-3 cells are shown in the right panel. Images were captured using an inverted light microscope {ZEISS Primo Vert (Jena, Germany)} at 10X. Total RNA prepared from PC-3AR and isogenic PC-3 cells (E-Cadherin and ZEB-2 n= 2 biological replicates with three technical replicates) was used to measure changes in expression of E-cadherin and ZEB-2. **C.** Logarithmically growing PC-3 and PC-3-AR cells (n= 3 biological replicates each with triplicate replicates) were transfected with RON-luciferase along with Renilla luciferase. 24h post transfection, cells were grown for additional 24h in media containing 10% serum and charcoal stripped serum. Cell lysates were prepared to measure luciferase activity. Data presented is an average+sd of all experiments. **D.** LNCaP cells (n= 4 biological replicates each with 6 technical replicates) co-transfected with RON-reporter plasmid and AR siRNA along with Renilla luciferase. 48h after transfection, cell lysates were prepared to measure RON promoter activity. Statistical significance of the data was analyzed using student's t-test and p<0.05 was considered significant (* = p ≤ 0.05; ** = p ≤ 0.01; *** = p ≤ 0.001).

To directly demonstrate the role of AR in regulation of RON, we examined the levels and expression of RON by stably overexpressing AR in AR negative PC-3 cells. Overexpression of AR in PC-3 cells caused significant decrease in RON expression (p=0.026; Figure 5A). Interestingly, reduced RON expression correlated with decreased ZEB-2 with no significant change in E-cadherin and morphological changes indicative of mesenchymal to epithelial transition (Figure 5B). Whether the observed decrease in ZEB-2 is causal or effect of RON or cross talk between RON and AR is unclear. We speculate that AR can reduce RON levels and thereby cause MET under normal growth (androgen proficient) conditions. In addition, we found that RON promoter activity significantly increased in PC-3 AR cells under androgen deprivation (AD) but not androgen proficient conditions compared to isogenic PC-3 cells without AR (Figure 5C). Furthermore, AR knockdown (using siRNA) in LNCaP cells increased RON promoter activity. These findings are consistent with Figure 4 and suggest that AR suppresses RON activation (Figure 5D). Taken together these observations suggest that AR could differentially regulate RON in a context-dependent manner. While under androgen replete conditions AR inhibits RON, however, under conditions of stress such as androgen deprivation it activates RON transcription. The precise mechanism of the switch from suppressor to activator and whether this is a transient or adaptive response requires further investigation.

### RON transcriptionally activates AR target gene c-FLIP

**Figure 6:**
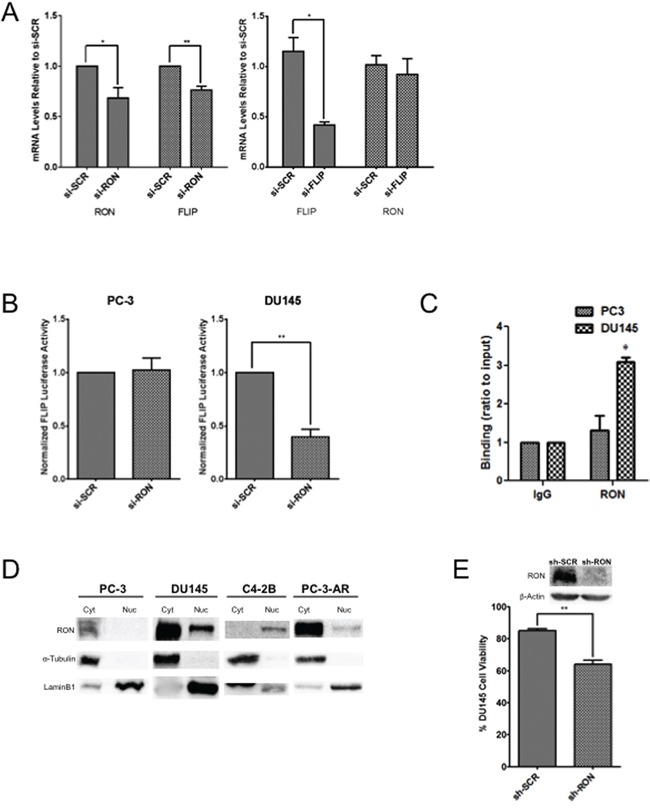
Nuclear localization of RON activates cFLIP transcriptionally **A.** Total RNA extracted from DU145 cells (n= 2 biological replicates with triplicate replicates) transiently transfected with si-RON or si-c-FLIP and scrambled control was used in real-time PCR using RON and c-FLIP-specific primers. **B.** Logarithmically growing PC-3 or DU145 cells (n= 3 biological replicates with triplicate replicates) were transfected with pGL3-c-FLIP reporter along with Renilla luciferase. 48h after transfection, luciferase activity was measured. Normalized luciferase/renilla activity was calculated with respect to scrambled siRNA. The data shown are average + sd of three independent experiments conducted in triplicate. **C.** DNA from IgG or RON-immunoprecipitated lysates from PC-3 (n=2 biological replicates each with triplicate technical replicates) or DU145 cells (n=2 biological replicates each with triplicate technical replicates) was amplified by real-time PCR using primers for the RON binding site on the c-FLIP promoter. DNA binding was calculated (in arbitrary units) by normalizing to input DNA. IgG was used as a negative control. Fold enrichment was calculated as 100*2^−(Ct[Target]-Ct[Input])^ and the amplification value from immunoprecipitated DNA was normalized to 10% input (p≤0.05). The data presented is mean+s.e.m from two indepdent experiments each with three technical replicates. Statistical significance was determined using two-sided t-test with no adjustment for multiple comparisons. **D.** Nuclear and cytosolic extracts prepared from PC-3 (n=3), PC-3AR (n=3), C4-2B (n=3) and DU145 (n=2) cells (all biological replicates) were probed for RON. α-Tubulin and Lamin B1 were used as loading controls for cytosolic and nuclear proteins, respectively. **E.** Percent cell viability (average+SD) of DU145 stable Scramble or RON silenced cells (n= 3 biological replicates) growing under androgen-depleted conditions for 120h from three independent experiments is presented. (* = p≤ 0.05; ** = p ≤ 0.01; *** = p ≤ 0.001).

Tumor epithelial cells survive in the tumor microenvironment by adhering to the extracellular matrix. Upon loss of adhesion, these cells normally die through detachment-induced apoptosis known as anoikis [34]. However, resistance to anoikis could lead to therapeutic resistance including emergence of CRPC. RON may promote tumor growth by inducing EMT and suppressing apoptotic signaling including anoikis. Anti-apoptotic and AR target gene c-FLIP is aberrantly expressed in human prostate tumors including CRPC; inhibition of c-FLIP sensitizes prostate cancer cells to apoptosis [35–37]. Previous studies from various laboratories including our own demonstrated significantly elevated levels of c-FLIP in PCA and CRPC [35]. The fact that RON is also elevated in advanced-stage PCA, indicates a possible signaling relationship between RON and c-FLIP. Therefore, we analyzed the impact of RON on the expression of c-FLIP and *vice versa*. Knockdown of RON reduced expression of c-FLIP with no change in protein levels in DU145 cells suggesting that RON induces c-FLIP possibly at the transcriptional level (Figure 6A left panel). However, knockdown of c-FLIP did not change RON expression (Figure 6A right panel). Based on these observations, we analyzed the transcriptional activity of c-FLIP in androgen independent cells under RON proficient and deficient conditions. RON-KD reduced c-FLIP transcriptional activity significantly in DU145 but not in PC-3 cells compared to non-targeted control (Figure 6B). Although number of possibilities exists, a notable difference between PC-3 and DU145 cells is status of tumor suppressor PTEN. DU145 cells are wild type for PTEN whereas PC-3 has mutated PTEN leading to constitutive activation of PI3K/AKT signaling [52]. We suspect the differences in regulation of c-FLIP between these cell lines may be attributed to the status of PTEN to prevent apoptosis by diversifying c-FLIP's upstream regulators. Although RON is a known receptor tyrosine kinase, the above findings suggest the distinct possibility that RON may transcriptionally activate c-FLIP in a context or cell-dependent manner. Though not extensively studied, RON has been reported to function as a transcription factor by binding to consensus sequence 5′-GCA(G) GGGGCAGCG-3′ depending on the context [10, 38]. Examination of the c-FLIP promoter sequence identified a putative binding site for RON in close proximity to transcription factor Sp1 at site +10. ChIP analysis showed binding of RON to c-FLIP promoter (C_T_ ∼29 to 31) in DU145 cells but not in PC-3 cells (Figure 6C). These observations correlate with nuclear localization of RON in DU145 cells but not in PC-3 cells (Figure 6D). We also observed nuclear RON in castrate resistant C4-2B cells (Figure 6D). Furthermore, the viability of RON-KD cells growing under androgen-depleted conditions was significantly reduced (Figure 6E). Given that c-FLIP is an anti-apoptotic factor, we examined apoptosis by analyzing levels of cleaved PARP under these experimental conditions. Interestingly, we did not observe significant changes in apoptosis as evidenced indicating that RON plays a major role in cell survival via c-FLIP rather than apoptosis (data not shown). However, we cannot rule out the possibility that it may play a role via autophagy or necroptosis. These findings along with data presented in Figure 5C, suggest that RON possibly plays a major role in survival of cancer cells under stress such as androgen deprivation.

## DISCUSSION

Although recent evidence shows that mice overexpressing RON under the control of prostate-specific probasin promoter develop PIN lesions suggesting RON plays an important role in prostate carcinogenesis, it has not been studied much in prostate compared to other tumor types [24, 25]. Here, we report for the first time the involvement of RON in castrate-resistant prostate cancer and its differential regulation by AR under androgen-proficient and androgen-deprived growth conditions. RON overexpression has been reported to enhance metastatic potential of mouse mammary tumors in the absence of ER-α [39]. Therefore, it is conceivable that elevated levels of RON observed in androgen independent cells in the absence of AR can contribute to greater metastatic potential and ultimately lead to androgen independence. Our data also implies that RON co-opts native AR signaling and activates some of AR downstream targets thereby promoting resistance to castration. Overall our results suggest that activation of RON can be a by-pass mechanism allowing for AR-signaling without native AR. However, the precise mechanism how RON causes EMT changes and castrate resistance is unclear. Given the published data showing association of mesenchymal genes such as N-cadherin with acquisition of EMT phenotype [40], we speculate that RON (in the absence of AR) can contribute to greater metastatic potential by promoting activation of N-cadherin ultimately leading to androgen independence. Alternatively, RON could contribute to castrate resistance by activating c-FLIP and other AR-target genes.

At the functional level, our data shows that RON plays an active role in EMT by altering mechanical properties of cells including cell adhesion and elasticity characterized by cytoskeletal reorganization. The obtained values of elasticity agree well with the published observations, usually falling into a range of 3–6 kPa [41–43]. Adhesion data are more difficult to directly compare since there is a substantial variability in the methods applied [44–46]. Actin dependent membrane protrusions act as critical determinants of EMT, therefore the disappearance of numerous filopodial structures upon RON-KD is in agreement with other parameters of RON's role in EMT. Changes in EMT markers correlated with these observations suggesting RON expression facilitates EMT in these cells. These data have important therapeutic implications given the involvement of androgen-induced EMT changes and cytoskeletal organization in the metastatic behavior of androgen independent prostate cancer cells.

Furthermore, we discovered that in addition to its receptor tyrosine kinase role; RON may function as a transcription factor to induce c-FLIP in a contextual manner. Although RON has been traditionally viewed as receptor tyrosine kinase, a recent report demonstrated that it could function as a transcription factor in bladder cancer cells [10]. Under stressful conditions including such as hypoxia and serum-starvation RON localizes to nucleus [10, 38]. While in the nucleus, RON functions as a transcription factor to induce expression of target genes including c-JUN and Bcl-2 [10, 38]. In this regard our results are consistent with these published reports.

While the relationship between RON and apoptosis evasion has been studied, the precise mechanism remains unknown. Though our data does not demonstrate the involvement of RON in apoptosis, the observation that c-FLIP is a downstream target of RON and their possible co-regulation is novel. We speculate that as a transcription factor RON may induce cell growth and as a receptor tyrosine kinase, it can promote EMT. It was reported that AR-regulated transmembrane protease serine 2 (TMPRSS2) contributes to pro-invasive EMT phenotype by activating the RON homolog c-MET [47]. Further, inhibition of TMPRSS2 suppresses prostate cancer metastasis *in vivo*. Interestingly, both LNCaP and C4-2B but not DU145 and PC-3 cells express TMPRSS2 [47]. Therefore, RON could be involved in promoting EMT and maintaining castration resistance via TMPRSS2.

c-MET, a close homolog of RON, is suppressed by AR in prostate cancer cell lines [48, 49]. Elevated expression of both RON and c-MET in various tumors including breast and colon is associated with poor prognosis, suggesting a critical role for RON signaling in cancer cell survival, migration, angiogenesis and therapeutic-resistance [20, 21, 50]. Specific inhibition of RON enhanced c-MET signaling leading to delayed tumor progression in a pancreatic cancer model [51]. It is possible that RON dimerizes with c-MET or another RTK known to promote tumorigenesis. Alternately, RON may act independently, but still similarly to c-MET owing to its sequence homology. Analysis of their independent or co-dependent and compensatory functions will clarify the individual roles of RON and cMET. In future studies, we will examine the role of RON isoforms and possible cross talk with c-MET in AR regulation including modulation of AR variants.

**Figure 7:**
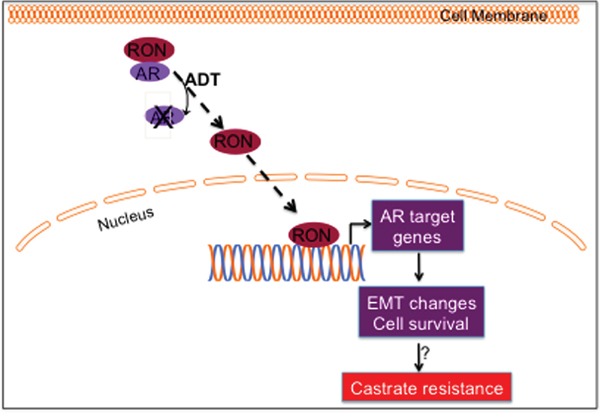
Hypothetical model During hormone responsive (HR) conditions, AR and RON exert a mutually inhibitory effect. We speculate that in the absence of AR or under conditions of androgen deprivation, RON translocates to nucleus and transcriptionally upregulates sub-set of AR target genes including c-FLIP to inhibit apoptosis and promote cell survival; influences EMT process by activating ZEB-2 and reducing E-cadherin. All of these events could contribute to progression to CRPC.

If one accepts that RON can contextually promote androgen signaling, administration of hormone-ablation therapy serves to aid and supplement RON's function and may even accelerate the onset of castration-resistant tumor growth. We speculate that elevated expression of RON could be a reason for prostate cancer transitioning into an aggressive, castration resistant state and possibly maintaining tumor aggressiveness. Based on these observations, we hypothesize a signaling model whereby RON contributes to castrate-resistance by functioning as a transcription factor to inhibit AR yet induce its downstream targets (which can induce EMT changes) including c-FLIP to promote cell survival (Figure 7). However, we do not rule out the possibility that RON can have differential effects on AR and its target genes in a RON level-dependent manner. Additional investigations including validation of these observations in additional cells and stable cells overexpressing RON are warranted to generalize these conclusions. Our data therefore suggests that RON merits serious consideration as a target for inhibition during hormone deprivation therapy.

## MATERIALS AND METHODS

### Cell lines

Prostate cancer cell lines LNCaP, PC-3, and DU145 were obtained from American Type Culture Collection (ATCC, Rockville, MD). BPH-1 and C4-2B cells were generously provided by Drs. M. Scott Lucia (University of Colorado, Denver, CO) and Thambi Dorai (New York Medical University, Valhalla, NY) respectively. These cells were grown essentially as described by us previously [52]. PC-3AR cells generated by Heisler et. al, were used [53].

### RNA and qPCR

Total RNA was extracted using TRIZOL and used to generate cDNA for gene expression experiments [52]. Expression of target gene mRNA transcripts was determined by Realtime PCR with gene-specific primers and SYBR-green PCR mix (Life Technologies). The primer sequences were: forward 5′-AGCCCACGCTCAGTGTCTAT-3′ and reverse 5′-GGGCACTAGGATCATCTGTCA-3′ for RON; forward: 5′-AAGCTGACTTCTTCTGGAGCCTGT-3 and reverse 5′-TCTCCTTGGCAGAAACTCTGCTGT-3 for c-FLIP′; forward: 5′-GGCACCCAGCACAATGAAGATCAA-3′ and reverse: 5′-AGAAGCATTTGCGGTGGACGATG-3′ for β-actin; forward: 5′-ACACTGCCAACTGGCTGGAGATTA-3′; reverse: 5′ TGATTAGGGCTGTGTACGTGCTGT-3 for E-cadherin′; forward: 5′-TAACCCAAGGAGCAGGTAATCGCA-3 and reverse: 5′-GTTTCTTGCAGTTTGGGCACTCGT-3′ for ZEB-2; forward 5′-GATTGAGCATGGCTCTCTATTC-3 and reverse 5′ GGTGAGATGTTCCAGGTTTAAG-3 for FKBP1; forward 5′-CATGTGATGTCTGGTCTGAAT-3 and reverse 5′-GACACAGCTCAACAAAGAAAC-3 for PMEPA1. The relative expression changes of individual genes were determined using comparative c_t_ method. Data is expressed as gene expression changes relative to β-actin control. Changes in expression of RON and c-FLIP were analyzed using Origene PCA II cDNA array HPRT 302 and 303 (for RON and c-FLIP expression respectively) containing different Gleason grade human prostate tumors.

### Generation of stably and transiently silenced RON cells

Logarithmically growing PC-3 or DU145 cells were transfected with non-target shRNA control or MST1R Sure Silencing shRNA (KH07170) using Lipofectamine 2000 according to the vendor's recommendation. 48h following transfection, cells were treated with 0.5 μg/ml puromycin for the selection of positive clones. The levels and expression of RON was analyzed by western blot and qPCR respectively using mixed population of puromycin-resistant cells. In transient silencing experiments, logarithmically growing DU145 and PC-3 cells were transfected with 25 and 50 nM ON-TARGETplus Human MST1R siRNA smart pool (Dharmacon) respectively using Lipofectamine 2000 according to manufacturer's recommendation. 48-72h after transfection, levels and expression of RON were analyzed by western blot and qPCR respectively.

### Cell viability experiments

DU145 stable Scramble or RON silenced cells were seeded in duplicate wells in a 24-well plate at 10,000 cells/well in 500μl media. 24 hours after seeding, media was changed to serum-free conditions. Viability of cells was determined by trypan blue exclusion at 48, 96 and 120h.

### Immunoblot analysis

Whole cell extracts were prepared from cells using 2X SDS-containing Laemmle buffer to determine the levels of proteins by immunoblot analysis as described previously [54]. Primary antibodies used include anti-RON (Santa Cruz Biotechnology; SC-322); anti-E-cadherin (Cell Signaling Technology, Inc., #3195) and anti-β-actin (Sigma-Aldrich; A5316). Nuclear and cytosolic proteins were prepared using “NE-PER Nuclear and Cytoplasmic Extraction Reagents” kit (Thermo Scientific; Waltham, MA, USA). Protein fractions were prepared for western blot using 6X SDS loading buffer. α-Tubulin (SC-5286; 1:1000) and LaminB1 (ab 16048; 1:5000) from Santa Cruz Biotechnology and Abcam were used as loading controls for cytoplasmic and nuclear fractions, respectively. The bound antibodies were detected by HRP-conjugated secondary antibody. Immunoreactivity was visualized using the ECL kit (Thermo Fisher Scientific, Waltham, MA) and digitally imaged using the Syngene G-Box (Syngene, Frederick, MD). The relative levels of individual proteins relative to β-actin loading control were analyzed with Gene tools software.

### Wound scratch assay

A monolayer of fully confluent PC-3 and DU145 cells stably silenced for RON was scratched to generate a wound. Cells were washed with PBS and fresh media was added. Pictures were taken and considered as 0h. Cells were monitored every 6h for the wound closure and photographs were taken.

### Analysis of nanomechanical properties of cells

Adherent PC-3 cells immersed in a culture medium were directly scanned with AFM in 55 mm uncoated petri dishes without any additional processing or immobilization. Cells from a single dish were imaged for up to 90 min without morphological signs indicating loss of their viability. Cells were scanned with a Nanoscope Catalyst (Bruker) AFM mounted on a Nikon Ti inverted epifluorescent microscope using the Peak Force Quantitative Nanomechanical Mapping (PF-QNM) mode (Bruker). Before AFM imaging, a light microscopic image was recorded for each cell. Scanning of a single cell took about 12 to 15 min. Electronic resolution of 30×30 to 50×50 μm square images varied from 64×64 to 256×256 pixels (x, number of points per line by y, number of lines). SCANASYST-AIR (Bruker) probes were used for imaging. The spring constant of the nominal value 0.02 N/m was determined for each probe with the thermal tuning. To determine cell boundaries, a cell shape and nanotopography was collected in height and peak force error channels, respectively. In parallel, the nanomechanical data consisting of cell elasticity and adhesion were captured in two additional channels. Nanomechanical parameters were calculated with Nanoscope Analysis software v.4.1 using the retrace images. Calculation of the elastic modulus followed the rules published by Sokolov assuming a high heterogeneity of cell surface properties (brush and rigidity) [55]. Additionally, we included adhesion forces in all the analysis. Calculations were performed based on the Sneddon model that approximates the mechanics of conical tip interactions with an object. A mode value of elasticity and adhesion for each cell was extracted from corresponding distribution histograms and applied in all the downstream statistical evaluations.

### Transient expression assays

The transcriptional activity of pGL3-ARE containing three repeats of PSA ARE in pGL3 reporter plasmid; AR promoter containing 1.7 kb 5′-flanking sequence; −503/+242 c-FLIP and 1.2 kb RON was measured by luciferase reporter assay. Briefly, logarithmically growing cells were transfected with respective reporter and Renilla luciferase plasmids using Lipofectamine 2000 as described previously [35]. 48h after transfection luciferase activity was measured by Dual-Glo Luciferase assay system (Promega Corporation). Luminescence was measured using the Promega Glomax 20/20 Luminometer and results were expressed as ratio of Firefly luciferase to Renilla luciferase.

### Phalloidin staining

Rhodamine conjugated phalloidin was used to visualize the impact of RON on alterations in F-actin. Briefly, logarithmically growing cells with or without RON knockdown were grown on coverslips in a 12-well plate and fixed in 3.7% formaldehyde (methanol free) for 10 min. Following permeabilization, cells were stained with Rhodamine-Phalloidin as per manufacturer's recommendation and examined under a confocal microscope. Images were acquired on a Sweptfield confocal system (Prairie Technologies, Middleton, WI) equipped with a Nikon Ti microscope. All images were taken with a 100X/NA 1.4 oil immersion objective. The images were captured on a Quantem 512SC EMCCD camera (Photometrics, Tucson, AZ).

### Immunohistochemistry

Tissue microarray containing different Gleason grade human prostate tumors were obtained from an IRB approved tissue repository at the University of Texas Health Science Center at San Antonio. Immunohistochemical evaluation of RON was performed essentially as described by us previously [35, 52, 56]. We used a Santa Cruz rabbit polyclonal antibody (sc-322) that has been validated and recommended for IHC in paraffin embedded tissues. Also the antibody has been validated in The Human Protein Atlas (www.proteinatlas.org.) We tested the antibody in several cell lines and human tissues including colon and breast with consistent results. We found staining patterns to be cytoplasmic in human tissue. Positive and negative controls were used with each staining run to identify problems with immunohistochemistry. All samples were stained at the same time with the same reagents. Total RON staining was scored as the product of the staining intensity (on a scale of 0–3) and the percentage of cells stained on a scale of 0-5, resulting in a scale of 0–8. Staining intensity was scored as follows: 0, none of the cells stained positively; 1, weak staining; 2, moderate staining intensity; and 3, strong staining intensity. Percent staining was scored as follows: 1, 20%; 2, 30%; 3, 60%; 4, 80% and 5, 100% cells stained. Wilcoxon (Mann-Whitney U) test was performed to determine if the mean ranks of RON total scores differed among tissues grouped by low Gleason of 4 or 6 (n=18) vs. High Gleason of 7 to 10 (n=10). The groups were also compared with a T test allowing for unequal variances with a Welch approximation with similar results but the non-parametrical test was considered the best fit for the data. Besides graphing the data into box plots (the line represents the media) it was also plotted as a histogram (data not shown).

## SUPPLEMENTARY FIGURES


