# Supplementary material for: Crosstalk between RON and androgen receptor signaling in the development of castration resistant prostate cancer
Source: Oncotarget. 2016 Feb 9;7(12):14048–63. doi: 10.18632/oncotarget.7287 (PMC4924697; doi:10.18632/oncotarget.7287)
Supplement: Supplementary file 1 [file oncotarget-07-14048-s001.pdf]

SUPPLEMENTARY FIGURES

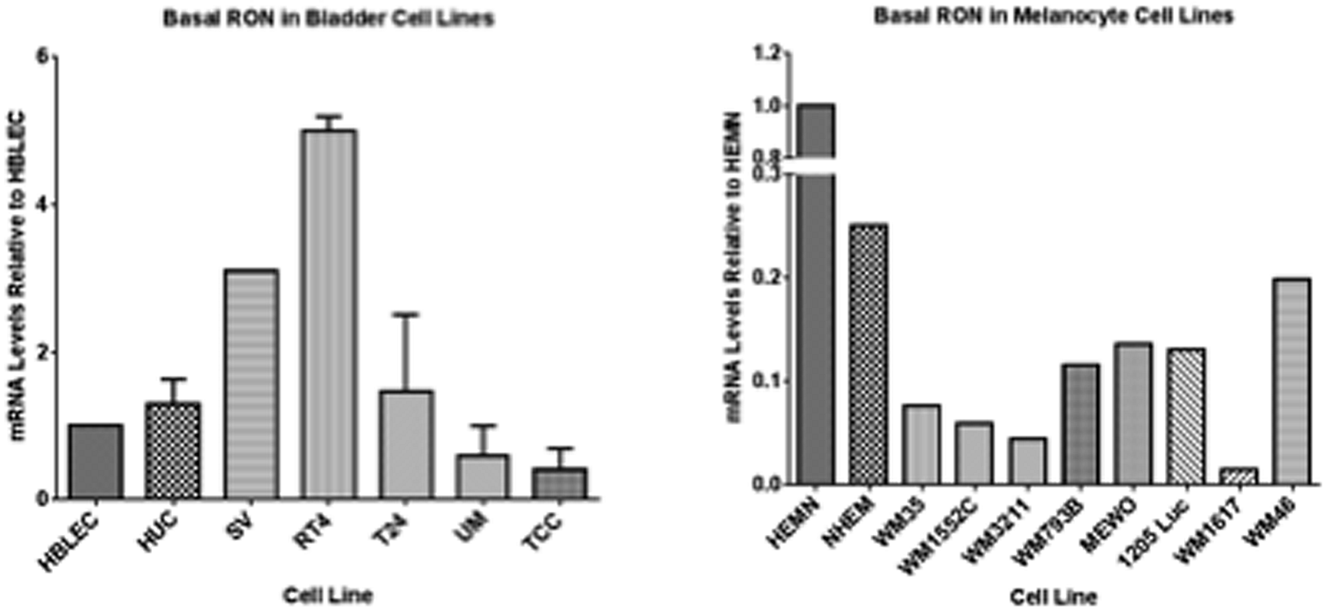

Supplementary Figure 1: Basal expression of RON in bladder and melanoma cell lines.

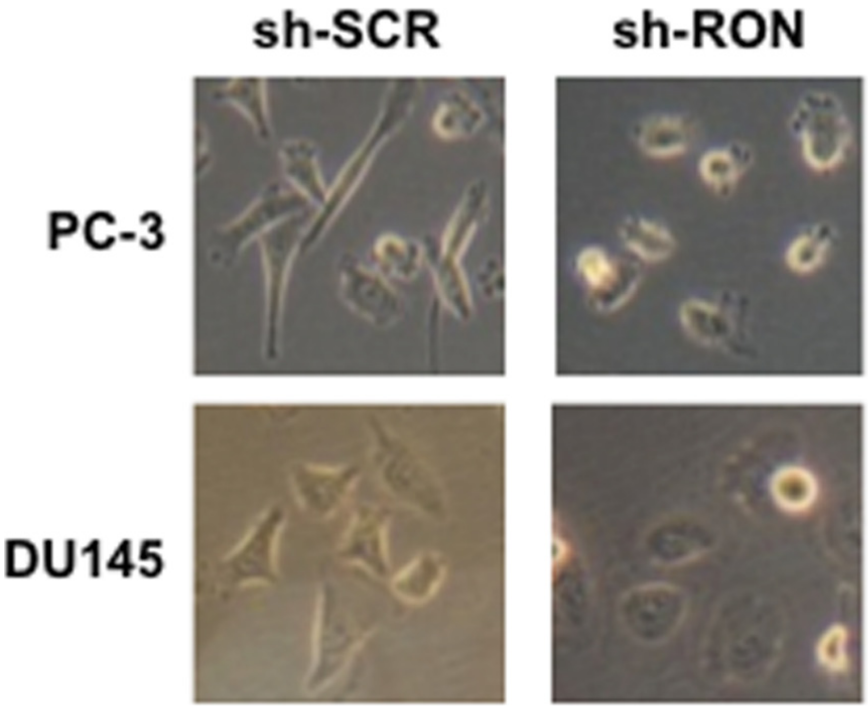

Supplementary Figure 2: Higher magnification phase contrast images of Morphological alterations associated with RON in prostate cancer cell lines.
